# Metaverse-based virtual reality experience and endurance performance in sports economy: Mediating role of mental health and performance anxiety

**DOI:** 10.3389/fpubh.2022.991489

**Published:** 2022-10-03

**Authors:** Zengsong Huang, Deok-Hwan Choi, Bingsen Lai, Zhicheng Lu, Haijun Tian

**Affiliations:** ^1^School of Physical Education, Jiaying University, Meizhou, China; ^2^Department of Physical Education, Woosuk University, Wanju-gun, South Korea

**Keywords:** metaverse sports arena, virtual reality sporting experience, endurance performance, mental health, performance anxiety, social comparison theory

## Abstract

Metaverse sports arena is gaining popularity globally that empowers virtual reality sporting experience through digital avatars. The main objective of the current study is to explore the impact of the Metaverse-based virtual reality sporting experience on the endurance performance of young Chinese athletes, with the mediating role of their mental health condition and performance anxiety. The study's participants mainly included Chinese athletes, especially the sample group is an accurate depiction of young athletes using a convenience sampling approach. SEM-AMOS statistical software was used for the analysis and validation of the proposed relationships. The study findings statistically validate that mental health and performance anxiety fully mediate the direct associations between virtual reality sporting experiences and the endurance performance of young Chinese athletes. Interestingly, the mental health condition of the young Chinese athletes imposes a greater impact on their endurance performance, in contrast to the adverse effects of their performance anxiety. The outcomes of the present research guide young athletes on the opportunities to enhance their virtual reality sporting abilities and boost their endurance performance. Policymakers can also build systems to dissolve physical and geographical barriers, reduce performance anxiety, and sustain mental health in virtual reality sporting events through the metaverse.

## Introduction

Experiences in virtual reality (VR) and augmented reality (AR) are being quickly commoditized and made available to the general public, including professional athletes. The increasing technical advancement of cyber-physical systems is driving this trend. There have already been a number of initiatives using VR technology to experience and appreciate activities that are either expensive, risky, or geographically unavailable (1). VR horse riding simulators, for example, are constantly being developed for educational purposes (2), and anyone can now safely participate in extreme activities such as mountain biking and skydiving by using VR (3). Athletes have been able to put themselves in the shoes of other Winter Olympic Games competitors by partaking in VR experience demonstrations provided by the local organizing committee for the 2018 Winter Olympics. Athletes with access to VR technology may now experience vicariously the performance of other athletes and interact with their friends who are physically present at a game from any location. Thus, VR technology may aid athletes in transcending the limitations imposed by the physical realities of their situations and speaking fluently in any setting. Moreover, VR is gaining popularity in the world of sports for the simple reason that it provides players with new experiences and motivates them to become more immersed in their activity. Therefore, using newly produced technologies has become an increasingly urgent obligation for sports marketers. In a physically isolated virtual environment, the user's sense of being in a given place or time period, as well as their emotions while there, may shift. A substantial amount of research has been conducted in the area of VR on the effects of experiencing a sense of presence in virtual environments. Numerous researchers, for example, have investigated the relationship between a sense of presence in a virtual environment and one's emotions (4). Despite the fact that psychometric approaches are commonly used by researchers to detect the influence of presence on emotional experience in virtual settings, few studies provide neurophysiological evidence of such affective experience. Despite the fact that researchers typically use psychometric tools to determine the influence of presence, this is not the case. In order to improve performance and gain a competitive advantage over other athletes and teams, the athletic world is rapidly adopting new technology. A better understanding of the perception-action loop is required for performance enhancement (4). VR is one kind of technology that has the potential to significantly alter the sports training industry (VR). The term “VR” (VR) refers to any visual-based computer simulation of a real or imagined world in which technology is used to both display and interact with the surrounding environment (5). VR is a universal term. VR experiences allow users to intuitively engage in real time with 3D computer-generated objects. Users may be amused, educated, or instructed by these models. The goal is to improve sensorimotor skills by using VR as a training tool instead of just explaining (for example, to improve tactical or decision-making skills) or making people laugh (6).

VR systems offer a number of potential benefits for athletic training, which may include the following: environments and scenarios can be standardized; augmented information can be integrated to mentor performance; and the environment can be adaptively altered to create a variety of competitive situations (7). Due to the high frame rate of head-mounted displays (HMDs) like the Oculus Rift (Oculus, Irvine, California, United States), it is now feasible to develop software for use in sports training. The current generation of head-mounted display (HMD) virtual reality (VR) sports training systems for the consumer market is mostly focused on video teaching, such as the American football-specific STRIVR product. This program allows users to observe prerecorded plays from an egocentric perspective, providing sportsmen with immersive familiarization without the need to attend instruction. Due to the stereoscopic 360-degree field of view (8), VR offers benefits over 2D video playback in this case. These benefits may be geared toward teaching athletes about the plays, which may boost their confidence, or they may be geared toward entertaining players (9). For the VR environment to induce a profoundly positive transfer, it must combine functional movement fidelity with meaningful action options and the proper sensory information (10). In a variety of sports, such as basketball free-throw shooting, rugby side-stepping (11), and fly ball catching, the use of virtual reality as a training simulator has shown varying degrees of effectiveness. The task used by Bieńkiewicz et al. (12) was free throw shooting in basketball. First, seasoned athletes fired on a virtual simulator, averaging 47.1% accuracy with terminal feedback after each shot. They then moved on to the real-court transfer test, where they obtained an accuracy percentage of 53.1% (13). The lack of evidence of training transfer or success in the VR environment may be attributable to the absence of haptic input while shooting a virtual ball, which prevents the experience from feeling like real shooting (14). When natural haptic cues are removed, perception and action are disconnected. This might lead to training modifications that are inappropriate for the circumstances.

Mental exhaustion may impair endurance performance in athletes by reducing their motivation to exercise (15), but its mechanism is seldom investigated and poorly understood. Mental weariness may reduce physical and cognitive endurance performance (16). Drive is a significant predictor of success in endurance sports, in which competitors are forced to push themselves to their physical and mental limitations in order to cross the finish line first (17). As a consequence, in this perspective, we analyze the link between mental fatigue, motivation, and drive in athletes, given that research often focuses on either one or the other but seldom studies all three concurrently (18). People often feel anxiety before partaking in critical tasks such as public speaking or a meeting with their boss. When anxiety is experienced just before or during an activity, it impairs working memory capacity, reduces self-confidence, and is harmful to performance (19). Many people, anticipating the unpleasant consequences of feeling anxious, try to reduce anxiety by calming down (20). However, lowering worried feelings may be difficult since high arousal is instinctive and attempts to conceal or disguise anxiety are often fruitless (21). The research on the impact of virtual sports experiences on athletes' mental health is limited and many of these studies are descriptive in nature. However, they are only concerned with the negative effects of low endurance performance. Meanwhile, the low endurance performance of young Chinese athletes and their exposure to virtual sports remains largely unexplored. The study on sports psychology does not provide conclusive solutions to issues such as the influence of virtual sports experience on Chinese young athletes with inadequate endurance. For the aforementioned reasons and the found vacuum in the existing literature, the current study intends to fill the indicated gap by examining the mediating impact of mental health and performance anxiety on the link between virtual sport experience and endurance performance. Therefore, in line with the gap highlighted and issues broached in the above study, the authors have planned to achieve the following research objectives: (1) To examine the role of Metaverse-based virtual reality sporting experience in determining endurance performance of Chinese athletes; (2) To examine the mediating role of performance anxiety in the relationship between the Metaverse-based virtual reality sporting experience endurance performance of Chinese athletes; and (3) To examine the mediating role of mental health in the relationship between the Metaverse-based virtual reality sporting experience endurance performance of Chinese athletes.

## Literature review

### Metaverse-based virtual reality sporting experience

Virtual Reality (VR) is a computer-generated environment with seemingly genuine sights and objects that immerses the user in his or her surroundings. Virtual Reality headsets or helmets are used to perceive this world (21). In the last decade, VR applications have been extensively used in both behavioral medicine (for conditions such as phobias, PTSD, and autism) and rehabilitation medicine (for conditions such as strokes, Parkinson's disease, and developmental disabilities). Notably, VR therapy has shown potential in treating a range of mental health disorders. Numerous conditions, including phobias, obesity, chronic pain, and eating disorders, have been extensively studied as prospective candidates for VR therapy (22). In recent years, the use of VR exposure therapy (VRET) to treat anxiety and depression has increased. According to a growing body of research, VRET is an effective treatment for the symptoms of anxiety and depression. This has led to the increased popularity of VRET. On the other hand, the bulk of current research on the impact of VR-based therapies on anxiety and depression focuses on VRET, while VR experience as a treatment for these conditions gets relatively little consideration. The phrase “VR experience” refers to the incorporation of VR capabilities with conventional experience equipment, such as treadmills and stationary cycles. Specifically, a number of brands of modern experience equipment have sensors that may wirelessly link to a user's game console or personal computer. This allows athletes to engage in rigorous physical experiences while playing VR games.

### Performance anxiety

Anxiety is a unique emotion that can be recognized by its high level of arousal, negative valence, lack of clarity, and decreased sense of control (23). The study conceives of anxiety as “a state of discomfort and/or physiological arousal in reaction to stimuli containing unexpected circumstances and the potential for unpleasant outcomes,” which is in keeping with what has been discovered in previous research. Anxiety may be induced by quite a few little threats, such as just being in close proximity to another person or quickly reliving a traumatic incident. Both of these scenarios can make people feel uncomfortable. Or they may be significant, such as the prospect of failure, humiliation, or even injury to one's physical self (24). Throughout the course of an athlete's life, the threats that might make them feel nervous will constantly change. These triggers were found in studies conducted by Serrander et al. (25). The existing body of research on anxiety has, for the most part, focused on a certain personality attribute referred to as “trait anxiety.” An individual's propensity to feel anxiety is reflected in their trait anxiety, which is related to neuroticism in that it indicates an individual's tendency to experience anxiety. Recent research has focused on state anxiety, which is a transient experience that everybody, at any moment, is capable of experiencing (26). Anxiety might be a personality attribute or a state of mind, but either way, the two are inextricably linked. But even though people with a high level of trait anxiety are more likely to experience state anxiety more often and to a greater degree than people with a low level of trait anxiety (27), most people experience state anxiety more than once a day. Performance anxiety is one of these stimuli. The majority of individuals get anxious before performing. It is rare to find a performer or athlete who is not nervous before taking the stage or the field. For a few, though, worry is incapacitating. These people have crippling performance anxiety.

### Mental health

The physical and mental demands placed on professional athletes are unlike those placed on any other group of people, and this may put them at a higher risk of developing mental health disorders or participating in risky behaviors (15). In addition, the most competitive years of a professional athlete's career sometimes overlap with the first stages of the development of mental health issues. The “workplace” stresses of a top athlete include increased public scrutiny through mainstream and social media; restricted support networks as a result of relocation; group dynamics in team sports; the possibility of early retirement due to injury; and the absence of support from family and friends. There are a variety of ways in which an athlete's mental and physical health, as well as their performance, may be negatively impacted by how they experience and respond to stress (18). There is a lack of understanding of mental health and its possible influence on sports performance. In addition, there is a common idea that seeking therapy is a sign of weakness. Both of these factors hinder many athletes from seeking assistance for mental health concerns (28). There have been efforts to publicize sport-related mental health research results in order to advance the preventative measures, identification, and early treatment of psychopathology in elite athletes. Despite these efforts, however, some sports regulatory agencies continue to minimize the significance of mental illness in this population. It would be a tragedy for these organizations if their best athletes were unable to get therapy for their mental health that was either prompt or effective, or if they did not feel comfortable discussing their struggles with mental health within the context of the workplace culture. A review found that top athletes' intense physical activity may have the opposite effect, trying to increase symptoms of anxiety and depression as a result of overtraining, injury, and burnout. Although there is a well-established link between physical exercise and mental health (29), the review found that top athletes' intense physical activity may have the opposite effect (30). Many studies show that people in this age group are more likely to suffer from mental health issues such as eating disorders and suicide (25). However, this may not hold true in every situation. The prevalence rates of mental health issues among professional athletes were found to be equivalent to those reported in the general population by researchers from Australia. They discovered that more than half of all professional athletes had at least one mental health condition. Due to low rates of official athlete mental health screening procedures (23) and players' perceptions of inadequate mental health care, retired elite athletes are at an increased risk for mental illness. This is connected to low rates of official athlete mental health screening protocols (24). As a result of the immaturity of sports psychiatry and its research base (26, 31), the mental health care that is provided for elite athletes may not take into account sport-related elements that make people more likely to have mental health issues or diagnostic or therapeutic issues that may be unique to this group. These factors include things like the fact that people who participate in sports are more likely to have problems with their mental health.

### Endurance performance

The integration of several physiological and psychological systems, working together to control exercise intensity in a manner that will either shorten the amount of time it takes or increase the amount of work it gets done, is required for successful endurance performance. One such factor is the virtual reality experience. Previous research has indicated that VR workout games aid physical rehabilitation (22), encourage motivation (32), and promote physical activity (33). By totally immersing users in a three-dimensional virtual environment (VE) *via* a head-mounted display (HMD), contemporary VR systems may enhance user experiences by allowing users to interact “from inside” the virtual world. By using avatars, which are digital representations of the users in the virtual environment, one may have a higher sense of presence—the experience of really being and acting inside the VE. This leads to a more robust and lifelike perception of the offered stimuli (34). This is achievable. A user's sense of presence, or the sense of being there and taking part in the virtual environment (VE), may be enhanced by using avatars, which are digital representations of individuals in a virtual environment (VE). As a consequence, the stimuli may be perceived as being stronger and more natural (35). Thanks to motion capture technology, VR systems can also accurately recognize a user's limb and body movements and concurrently send them to the avatar's virtual representation. Previous research has been conducted as a result of the synchronization of visual and proprioceptive information, which has been proved to induce the illusion of inhabiting a virtual body. Users adopt the identity of the avatar as a kind of virtual self-expression, which lowers their anxiety and boosts their mental health and resilience (36). VR experiences might be used to treat mental health conditions like anxiety and depression since VR devices are now compatible with conventional exercise equipment like bikes and treadmills. Unfortunately, there is a dearth of thorough research on the impact of VR experiences on outcomes linked to anxiety and depression. As a result, the goal of this study is to examine the information already available about how exercising in VR affects anxiety and sadness. The most convincing data about the potential benefits of VR experiences will be found, gathered, and analyzed in this pilot research. Exergames may be made with creative engagement mechanisms thanks to VR. VR training games have been shown to be helpful for physical rehabilitation, increasing motivation, and encouraging physical exercise (7). By completely immersing users in a three-dimensional virtual environment (VE) *via* a head-mounted display, contemporary VR systems may improve user experiences (HMD). Avatars, which are digital representations of users in the virtual environment, can be used to achieve a greater sense of presence—the sensation of truly being and acting within the VE (37). As a result, the stimuli are seen as being stronger and more realistic (18). Additionally, when VR systems and motion capture technologies are used together, the user's limb and body motions may be precisely recognized and synchronized with the user's avatar. According to prior research, this synchronized connection between visual and proprioceptive information may provide the impression that a person has a virtual body. Proprioceptive and ocular information may be combined to produce this illusion. Because of this, users take on the avatar as a kind of digital alter ego, which lowers their anxiety, improves their mental health, and makes them stronger.

### Theoretical framework and hypothesis development

In humans, endurance performance ability is characterized by a considerable degree of inter-individual variance in both the general population and even in well-trained, athletic people (38). This diversity may be seen in both short-term and long-term performance. Historically speaking, this ability was generally evaluated by exercise scientists making use of physiology. The concentration of attention of an endurance athlete may have a substantial impact on the athlete's perception of the amount of effort being exerted, their ability to regulate their pace, and their physiological markers of performance. VR is an example of a technology that has the ability to encourage more people to participate in sports and improve their endurance performance (VR). Amateur athletes and leisure exercisers now have a practical option to employ VR throughout their training and workouts due to recent advancements in consumer technology that have made it more accessible and more affordable. VR is a technology that allows users to interact with and feel fully immersed in a virtual world (39). In VR-based exercise, interaction is often performed through an effort interface, such as a treadmill or ergometer, wherein the person's activities on a machine are converted into movement in the virtual world. These interfaces include the Virtuix Omni and the Virtuix Omni treadmill, among others. As described by Qian et al., research has been conducted on a variety of sports and physical activities (37). These activities include walking and running, cycling and rowing (40).

The use of VR has the potential to affect a variety of outcomes, including performance as well as physiological and psychological consequences that are detected simultaneously or post exercise (33). There is some evidence to show that the use of VR may improve the amount of physical effort that one experiences while exercising, as well as the length of time that one stays committed to an exercise program (34). It is possible for other people to be present in a virtual environment using the technology that is currently available for VR, even if they are physically situated in a different part of the world. This social component may have a significant impact on how individuals react when engaging in VR-based physical activity. For instance, Knicker et al. (41) evaluated the performance of rowing in a virtual environment and compared it to the performance of rowing in the same setting while the rower was either alone or with a teammate present. Participants who rowed with a partner had a higher average heart rate and covered a greater distance than those who rowed alone. Both the theory of social facilitation and the theory of social comparison may be used to explain why certain people do better when they are in the company of other people. However, such arguments are often applicable only in settings where there is no competition (35). Therefore, the study intends to test that:

*H1: VR sporting experience of athletes has significant impact on the endurance performance*.

A competitor's level of difficulty in a VR-based competitive setting has the ability to affect both performance and motivation. Individuals have a natural inclination, according to the social comparison hypothesis, to assess their own performance in relation to the performance of others in their surrounding environment (36). Behm and Carter (42) conducted study that did not utilize VR and involved riding a stationary bicycle. Individuals were compared based on their performance while cycling with either a highly fit or less fit riding buddy. Physiological and subjective exertion were used to evaluate performance. Despite being encouraged to maintain a moderate heart rate during the ride, individuals partnered with a high-fitness partner reported substantially greater heart rate and perceived effort levels than those placed with a low-fitness companion. On the basis of these statistics, it seems that the participants compared their own performance to that of their spouse and then made any required modifications to match their spouse. It is still unknown what effect social comparison might have on performance in a competitive situation when compared to an opponent whose level of competence differs (43). According to sports physiology studies, the bulk of these performance decreases may be linked to mental tiredness, which is believed to be the outcome of persistent, high-intensity mental activity (44). According to the psychological model of endurance performance (45), perception of effort is the “primary exercise stopper” (46), and perception of effort during the execution of a physical activity may be influenced by a variety of circumstances, including the induction of mental tiredness beforehand. This concept suggests, in example, that the perceived difficulty of a physical effort may be altered by a variety of factors, such as mental fatigue.

According to the findings of research that is relevant to cognitive neuroscience and psychology, certain regions of the prefrontal cortex play an essential function in the regulation of effortful control (32). Specifically, the anterior cingulate has been linked to the feeling of exertion (47), as well as the decision to invest further effort, and higher prefrontal cortex activity has been seen when people anticipate the need to expend mental effort on a task (48). In line with this notion, it has been shown that the level of exertion that participants in an endurance exercise are required to put out in order to complete the activity results in an increase in the amount of prefrontal brain activation (49). It is important to take note of a decrease in activation that often occurs just before the failure of a task that is being performed while engaging in strenuous cycling exercise (50). This provides evidence that the prefrontal cortex must maintain a certain level of activity in order to successfully perform a challenging task (51). This demonstrates that previous mental exertion creates a rapid rise in the perception of effort, which, when seen from the point of view of mental weariness, results in the completion of a job before it is completely finished (52). In the context of this idea, the first challenge is often referred to as the mental weariness or cognitive fatigue task. In light of this, the purpose of the research was to establish whether or not:

*H2: VR sporting experience of an athletes has significant impact on the mental health*.

It is useful to evaluate if the efficacy of an intervention extends beyond performance and, for instance, may be helpful to the mental health of an athlete. An intervention involving psychological skills may not result in immediate performance improvements; however, the athlete may be satisfied with the session and feel calmer or less stressed as a result of it. If this is the case, it is helpful to determine if an intervention's efficacy extends beyond the subject's performance. Frequently, if we want to perform to the best of our abilities, we must practice self-control and intentionally use mental effort in order to do something significant. Only then can we aspire to reach our full performance potential. For example, for a college student to get good grades, he or she must put in a lot of time studying and mental effort to overcome any internal (such as task-induced ennui) or external (such as peer-suggested alternative behaviors) barriers to achieving goals. For example, a student must devote a substantial amount of time to studying in order to get high grades in college. The same is true for a cyclist, who must exert mental effort in order to resist the temptation to ride at a slower pace despite her aching body. This is the case despite the fact that her body is beginning to hurt. Therefore, the efficient self-regulation of human performance typically requires the application of mental effort. In spite of (or maybe because of) the pervasiveness of the concept of mental effort, it has been very difficult to establish a working definition of the word (53). As a direct result, the purpose of the research was to establish whether or not:

*H3: Mental Health of athletes has significant impact on the endurance performance*.

Anxiety is a mental illness characterized by anxious thoughts, tense sensations, and physical changes including elevated blood pressure (40). A mixture of these symptoms may also be associated with anxiety. Anxiety disorders are characterized by persistent intrusive thoughts or worries, as well as physical symptoms include shaking, sweating, a high heart rate, or dizziness (33). Additionally, psychological disorders may cause vertigo. The use of virtual reality in the treatment of anxiety may improve its acceptance, effectiveness, and comfort. Virtual reality exposure therapy, or VRET for short, enables individualized, gradual, controlled, and immersive exposure. It is straightforward for therapists to administer, and patients find it more pleasant than *in vivo* or virtual exposure. Multiple unique types of VRET exist. Medication, healthy lifestyle modifications, mindfulness practices, and cognitive-behavioral therapy may all be used to treat anxiety and depression. Symptoms of anxiety and/or sadness are often alleviated by therapies that are customized to the patient's needs (40). There has been enormous public interest in the use of cutting-edge technology for health promotion up to the present. VR is without a doubt the most fascinating and technologically sophisticated of the upcoming technologies that have the potential to assist in the treatment of anxiety. Virtual reality (VR) is a computer technology that gives artificial sensory experiences, including visual, auditory, tactile, and olfactory sensations, and enables the user to control objects inside the generated virtual environment (34). Using virtual reality, the user may also traverse time and space inside the constructed virtual environment. Non-immersive VR uses interfaces such as flat-screen TVs or computer monitors, in addition to keyboards, gamepads, and joysticks. Immersive VR, on the other hand, often utilizes head-mounted displays, body-motion sensors, real-time graphics, and complicated interaction devices (such as specialized helmets) to create a completely virtual environment for the user. This creates a sensation of immersion inside the virtual world for the user. Immersive VR reproduces a virtual setting to a higher extent than non-immersive VR. In non-immersive VR, players won't feel as though they are “really there” as they would in immersive VR (36). My almost four decades of experience as a clinical psychologist at a university mental health clinic have kept me devoted to the treatment of people with severe performance anxiety. The primary reason for my participation is because I have spent a great deal of effort to aiding these people. Each year, a number of clients seek my assistance because their crippling performance anxiety threatens to end their academic or professional career because they are unable to speak in required classes, fail professional licensure exams, or experience writing blocks that prevent them from finishing their thesis. Aspiring musicians and college athletes suffered greatly from anxiety, and as a result, they performed far below their potential (41). As a result, we may infer that using VR to engage in a sporting event may assist athletes in overcoming performance anxiety. Consequently, the purpose of the investigation was to discover whether or not:

*H4: VR sporting experience of athletes has significant impact on the Performance anxiety*.

One of the primary areas of focus for study in the field of sports psychology continues to be anxiety and the implications it has on athletic performance (54). Anxiety may be characterized as an unpleasant emotional state that might emerge when one is confronted with potentially dangerous or stressful circumstances (55). Due to the fact that athletes participating in competitive sports are required to perform well under pressure, athletic tournaments may be seen as potentially hazardous evaluative circumstances, and as a result, they may likely trigger increased levels of anxiety. According to Martens et al. research (56), anxiety is a multidimensional construct that is made up of two primary components: cognitive anxiety (i.e., worrying thoughts about one's performance) and somatic anxiety (i.e., an individual's perception of his or her own physiological arousal, such as nervousness, tension, or heart rate). Anxiety has the potential to have an effect on a number of different facets of the sporting world. For instance, anxiety has been linked to quitting sports activities (57), experiencing less enjoyment when engaging in sports, and having reduced performance (58). The purpose of this research is to investigate the impact that performance anxiety has on the endurance performance of athletes participating in sports. The majority of studies (59) have indicated that anxiety has a detrimental effect on athletic performance, which has also been shown across a variety of athletic disciplines. For instance, it has been shown on several occasions that anxiety seems to be a significant component that may hinder performance in a variety of sports and activities, such as soccer penalty kicks (60), rock climbing (61), golf putting, or table tennis. However, the underlying mechanisms that are responsible for the negative impacts that anxiety has on athletic performance have not been thoroughly examined as of yet, and as a result, it seems to be of the utmost importance to uncover aspects that might possibly have an effect. Consequently, the following hypothesis were the focus of the research:

*H5: Mental Health of athletes has significant impact on the endurance performance*.

One meaning of the term “metacognition” is “thinking about thinking,” also known as “thinking about metacognition.” According to Perry et al. (27), the term “metacognition” refers to an individual's knowledge and cognitions about the events that occur during cognitive processing. It's possible that an individual's degree of metacognition may be inferred based on how well they understand what they know and how they might put that knowledge to use in order to manage their behavior (62). Not only does metacognition require the establishment of conscious goals, but it also involves the activation of strategies (in the form of thoughts and actions) to attain those goals. In other words, metacognition entails both the setting of goals and the activation of strategies. It is also very important to point out that while self-regulation and metacognition have different origins in the area of psychology, metacognition is recognized as a key component of effective self-regulation. This is a very important distinction to make. In light of this, Rhodes (29) place an emphasis on a “conceptual core” (p. 404) that connects self-regulation with metacognition. This fundamental component encompasses both the monitoring of one's thoughts and actions, as well as the engagement in activities designed to achieve control over both of these elements of one's behavior. In light of this, the next section will make an attempt to shed some more light on the ways in which endurance athletes monitor and regulate the thoughts and behaviors that are essential for effective pace-regulation.

The most obvious symptom of mental tiredness is a decline in one's cognitive performance. This kind of fatigue is brought on by indulging in intellectually hard tasks for lengthy periods of time (28). Participants in mentally challenging exercises such as the “Stroop task” are required to stifle their initial response in order to generate the appropriate response and find the right answer. They provide the perception of being challenging and laborious, which, in the end, results in a reduction in the user's cognitive efficiency and performance. It would seem that the duration of the task is rather crucial, since research has shown that activities lasting <30 min do not have any negative influence on later exercise performance; nevertheless, they may impair cognitive function (30). Ego-depletion is a phenomenon that is similar to mental tiredness in that it implies an expected reduction in performance after a rigorous effort that mostly requires inhibition. The phrase “ego-depletion” alludes to this phenomenon. On the other hand, the results of a recent meta-analysis demonstrated that short-term “ego-depleting” activities did not have any impact on subsequent endurance exercise performance. Some of the “side effects” that are associated with mental tiredness include a lack of energy, increased fatigability and feelings of lassitude, lower sensations of motivation and attention, and changes in perception and mood (63). As a consequence of this, it has been hypothesized that the effects of mental exhaustion may have twofold repercussions: first, it may hinder performance by increasing feelings of exhaustion, such as “I cannot do it, I am exhausted,” and second, it may cause an underestimation of the significance of succeeding at a particular task, such as “I do not feel like doing it, it is not worth it.”

On the other hand, the “strength of self-control hypothesis” postulates that activities that are draining on one's ego deplete a single global metaphorical strength that has a finite capacity and, as a result, inhibits further performance. This hypothesis is in contrast to the “ego-draining activities hypothesis,” which postulates that activities that are draining on one's ego do not have this effect (61). Despite this, the hypothesis that performance decreases are driven by the depletion of energy sources in the brain (such as glucose) has been disproved in a manner that is sufficiently credible. Perry et al. (27) makes a compelling case that the impacts of cognitively taxing activities need to be taken into account in light of an individual's decision to allocate resources in accordance with the level of significance they assign to the activity at hand. In light of this, our objective is to explore the effects of mental weariness by analyzing the dynamic between an individual's perception of the amount of work they are putting in and the degree of motivation they possess (29). The extent to which an athlete is motivated to perform well is directly proportional to the degree to which they feel their efforts will be rewarded and the degree to which they place a high value on those benefits (51). Therefore, mental exhaustion has the potential to impair driving in a number of different ways. This has been explained from a neurophysiological aspect by the existence in the brain of two distinct systems that are involved in the regulation of behavior: a mental inhibition system and a mental facilitation system. These systems work in conjunction with one another to regulate behavior. The prefrontal cortex is the region that contains both of these systems (60). The first one makes the athletes feel as if they are working harder, which in turn causes them to move more slowly. The second one, on the other hand, makes them feel more driven toward a reward, which in turn causes them to move more quickly (56). It is true that mental fatigue produces an increase in perceived effort, and researchers have found that it also stimulates the brain regions involved in the inhibitory system of athletes. Mental tiredness has been shown to alter brain areas involved in the cognitive part of central motor command as well as the facilitative system that ordinarily motivates athletes into action. This shows that mental tiredness may play a role in deactivating the mechanism that would typically inspire athletes to take action. It is therefore likely that mental weariness may have an effect on motivation because it has a negative impact on preparedness to undertake physical exertion in order to gain a reward. Despite the fact that this has not been proven in endurance athletes, it is likely that mental weariness may have an effect on motivation. It is interesting to observe that top cyclists seem to have a stronger tolerance to mental fatigue than their competitors who have not trained. This gives them a competitive advantage (23). As a direct result, the purpose of the research was to establish whether or not:

*H6: Mental health of athletes mediate the relationship between the VR sporting experience and endurance performance*.

Anxiety, despite the fact that it is unpleasant and upsetting, may have positive effects on the way a person responds in certain circumstances. For instance, people who feel apprehensive a significant amount of time before an event may find that it motivates them to exert effort and prepare *via* a process that is known as defensive pessimism. This kind of pessimism is known as defensive pessimism, and it arises when a person has pessimistic expectations about the course of future events. This leads the individual to make more efforts to protect themselves from possibly adverse consequences and to prepare more completely (31). According to research that is relevant to this issue, threat assessments may not necessarily be harmful to performance, but they could cause individuals to work harder on things that are simple or that they have mastered. In a manner similar to this, the Yerkes-Dodson law illustrates the connection between anxiety and performance by depicting it as having the form of an inverted U. An anxiety level that is either very low or excessively high is damaging, but an intermediate amount of anxiety may improve motivation when engaging in tasks that require endurance or tenacity (64). But worrying too much right before or while doing a task can make it harder to think and do well, especially for people who aren't experts in the field. This is especially true for those who have not prepared well for the activity. Both the capacity of one's working memory and their ability to comprehend information are impaired when one is anxious. People who are anxious are unable to focus on the task at hand because they use their working memory for tasks that are not useful, such as worrying and dwelling on problems (65). An aversion to danger and a lack of self-confidence are two examples of the negative effects that anxiety may have on motivational systems. Recent studies have shown that being in a state of anxiety may have an adverse effect on a person's self-efficacy, which can be defined as one's confidence in their own capacity to do a certain task effectively (66). A lack of confidence, on the other hand, has a substantial influence on both decision making and behavior. Anxious negotiators, for example, are more likely to make bad initial offers, leave the table fast, and earn a smaller profit than neutral state negotiators. This is because anxious negotiators experience higher levels of physiological stress. These results are the result of the negotiator's having a low degree of self-efficacy, which is the cause of the problem (31). People who suffer from anxiety are less confident in their own ability to make sound decisions, so they look for guidance from outside sources and tend to place a greater amount of weight on that guidance, even if it is patently unsound. In a related vein, people who suffer from anxiety are less confident in their own ability to solve problems. Exercising in VR can help with mental health problems like depression and anxiety. This is primarily due to the fact that VR systems may now be connected to regular exercise equipment like bikes and treadmills. VR e experience effects on outcomes linked to anxiety and depression are not well understood. In light of this, the goal of this study is to review the data on how exercising in VR affects anxiety and depression. In order to improve anxiety and depression outcomes, the strongest evidence will be found, compiled, and evaluated in this experimental investigation. The purpose of this effort is to provide the foundation for more study in this area. VR's development has made participatory activities possible. VR training games have been shown to improve motivation (41), promote physical activity (37), and support physical recovery. Modern VR systems may enhance user experiences by allowing interaction from “within” the virtual world by fully immersing users in a 3D VE *via* a head-mounted display (HMD). Because of this, the point of the study was to find out whether or not.

*H7: Performance anxiety of athletes mediate the relationship between the VR sporting experience and endurance performance*.

## Methodology

Exploratory research was conducted, and the quantitative data collection technique was used. The participants in this research are young Chinese athletes whose ages range from 12 to 18 years. This sample population would provide a realistic representation of young athletes. Data was collected by using a survey-based methodology. Additionally, the suitability of the sample must be verified in order to get a representative sample. Additionally, sampling is a technique that allows researchers to make judgments about a group without looking at every individual. Convenience sampling was employed in this study to choose a representative sample from the whole population. In essence, convenience sampling entails gathering information from members of the population who are easily available and willing to take part in this study. Since the self-administered questionnaire has a number of benefits, including low cost and quick response, it is utilized to collect the data. Respondents must choose either “strongly disagree” or “strongly agree” for each of the closed-ended items in the survey. Young Chinese athletes were given access to 400 questionnaires. Information was gathered from China's four major cities (i.e., Shanghai, Shenzhen, Guangzhou, and Beijing) Out of the 400 questionnaires sent, 145 were complete and appropriate for study. In addition, the G^*^Power software, version 3, was used in order to validate whether or not the sample size was enough based on a number of statistical considerations. SEM-AMOS statistical software was used for data analysis. It is also known as software for causal modeling and covariance analysis. AMOS is a tool for modeling structural equations graphically (SEM). In quantitative modeling, structural equation modeling is the most popular technique. It is most popular because of the underlying theory's sophistication. Moreover, it has the potential to address significant substantive questions.

### Measurement

In the current study, the questionnaire was designed using the five-point Likert scale. It is appropriate for the collection of data from a large population, especially with highly sensitive issues. For the collection of data, survey methodology is used. Survey-based methodology is appropriate for the collection of data from the respondents because it is easy to distribute and collect the data for the analysis. Thus, the current study used a survey-based methodology for the data collection. The scales were selected from the different research with careful analysis of their face validity and reliability in previous research. A comprehensive literature study of the relevant constructs was conducted before the measuring items were developed. Four experts were requested to evaluate the questionnaire's content validity in order to make sure it was efficient and easy to understand. On a Likert scale with a range of 1–5, where 1 means strongly disagree and 5 means strongly agree, the results were added up. For the measurement of virtual reality sporting experience, we used 3 items scale developed by Livneh (65) (e.g., 1. Searching the XYZ Web site in this way “gets me away from it all). We measured mental health with the help of three items scale developed by Planey et al. (66) which had a good reliability and validity in previous researches (e.g., I often feel calm and stable). For the measurement of performance anxiety, we relied upon the two items scales developed by Mathwick and Rigdon (67), having good reliability in previous researches (e.g., When difficulties arose I calmly considered how I could continue the task). Lastly, we used 8 items scale developed by Planey et al. (66) for the measurement of sport endurance performance. The scale had good reliability in previous researches. (e.g., I have a good team commitment).

### Process of data collection

This section demonstrates a detailed data collection process. The respondents of current study were the young athletes of China. For the sample selection in current study, non-probability sampling technique was used. Convenient sampling was used for the selection of the sample. It is a method of collecting the data from the conveniently and easily available sample. Before collecting the data and distribution of questionnaire, consent of the respondents was taken. To the respondents with the positive response questionnaires were distributed. We explained the questionnaire briefly to the respondents to get familiarity and the clarity of the questions. For the studies of mental health expected response rate is 50% according to the previous researches, therefore, for the adequate set of useable data we distributed 400 questionnaires. Out of 400 we received back 210 and the usable data was of 145 (Table 1). Data was collected from the four top metropolitan cities in China (i.e., Shanghai, Shenzhen, Guangzhou, and Beijing).

**Table 1:** Response rate.

**Total distributed**	**400**
Received back	210
After removing outliers	189
After missing value removal	145
Total usable data	145

## Data analysis

### Analytical strategy

The data was examined in four phases. First, we did confirmatory factor analysis to verify the reliability and validity of the measurement items for all the measures Anderson and Gerbing (68). After the development of discriminant and convergent validity, the hypothesized structural model was evaluated with the use of structural equation modeling (SEM) (68). For assessing the model fit, SEM incorporates a variety of multivariate approaches which are confirmatory rather than exploratory. Over standard multivariate approaches, SEM provides three key benefits. Firstly, it estimates the unobserved variable (latent variable) with the aid of observable factors. Secondly, it specifically examines the measurement inaccuracy. Thirdly, it evaluates the model where a structure may be appraised and enforced (69). Measurement error is mistakenly neglected by most of the multivariate approaches since they do not model it explicitly. Conversely, SEM assesses the variance of error for both dependent and independent variables Glomb and Liao (70). We looked at both the direct and indirect effects of virtual reality sports experiences on endurance performance in sports.

### Descriptive statistics

Table of descriptive statistics shows the mean and the standard deviation of the variables under study (71). Moreover, it explains the relationship among variables. In our study all variables have the mean values between descriptive statistics and correlation (Table 2).

**Table 2:** Descriptive statistics and correlation.

	**Mean**	**SD**	**1**	**2**	**3**	**4**
VRS	3.23	1.19	1			
MH	3.41	1.25	0.68^**^	1		
PA	3.62	1.70	0.75^**^	0.56^**^	1	
SEP	3.42	1.12	0.71^**^	0.72^**^	0.66^**^	1

** 0.001 < p < 0.01.

All variables have significant positive relationship with the dependent variable. Therefore, the data is appropriate for the further analysis.

### Confirmatory factor analysis

We measured the construct with a well-known instrument. In order to check the dimensionality and reliability, confirmatory factor analysis (CFA) was performed. We had four factors in total: virtual reality sporting experience, mental health, performance anxiety, and sport endurance performance. Initially, a single-factor CFA was conducted by loading all the items of four measures onto one factor Anderson (68). One factor/single-factor CFA did not produce a good fit (Table 3). Then we performed the four factor CFA by loading all items on their respective measures. A four-factor CFA produced a good fit.

**Table 3:** Model fit indices.

**Models**									
		χ^2^	**Df**	χ^2^**/df**	**GFI**	**NFI**	**TLI**	**CFI**	**RMSEA**
Model 1	One factor CFA	294.04	104	2.81	0.77	0.55	0.81	0.72	0.09
Model 2	four factor CFA	193.06	98	1.97	0.91	0.92	0.95	0.96	0.06

With the four-factor CFA, high factor loadings were exhibted by all factors 0.67–0.86 (72). All factors of VRS have significant loading i.e., 0.78, 0.76 and 0.70. Similarly, all factors of PA have factor loadings higher than 0.6 i.e., 0.86 and 0.68, respectively. All three items of mental health have loading >0.7 i.e., 0.80, 0.79 and 0.80, respectively. Similarly, All eight items of SEP have loading >0.70 (See Table 4). Therefore, all items of the constructs were used for the data analysis.

**Table 4:** Model fit indices.

			**Estimate**
vrs1	<–	VRS	0.78
vrs2	<–	VRS	0.76
vrs3	<–	VRS	0.70
sep1	<–	SEP	0.71
pa1	<–	PA	0.86
pa2	<–	PA	0.68
mh1	<–	MH	0.80
mh2	<–	MH	0.79
mh3	<–	MH	0.80
sep2	<–	SEP	0.71
sep3	<–	SEP	0.79
sep4	<–	SEP	0.74
sep5	<–	SEP	0.78
sep6	<–	SEP	0.78
sep7	<–	SEP	0.76
sep8	<–	SEP	0.72

The AVE (Average variance Extracted) of all factors is >0.5, explaining the convergent validity satisfactory. After it we established the discriminant validity of all constructs i.e., virtual reality sporting experience, mental health, performance anxiety and sport endurance performance (see Table 5).

**Table 5:** Reliability.

	**Reliability**	**AVE**
VRS	0.79	0.56
SEP	0.89	0.57
MH	0.84	0.64
PA	0.75	0.60

Four variables showed high internal reliability and consistency with Cronbach's alpha values (see Table 6) of 0.72 for VRS, 0.89 for SEP, 0.84 for MH and 0.75 for performance anxiety,—all >0.7 the recommended value (73). On diagonal AVE and below are the values of squared correlations.

**Table 6:** Construct reliability and validity.

	**1**	**2**	**3**	**4**
VRS	0.56			
MH	0.47^**^	0.64		
PA	0.57^**^	0.31^**^	0.60	
SEP	0.51^**^	0.52^**^	0.44^**^	0.57

Using a cross-sectional study design, the same questionnaire was used to gather data throughout a particular time period; hence, the possibility of common method bias exists (74). Harman's one factor method was used to test for CMB existence. Harman's one-factor approach was utilized to test for the existence of CMB in the first factor. The first component explained less than half of the variation, implying that CMB did not exist in the data.

For the multicollinearity test we check the values of variance inflation factor (VIF). High values indicate the existence of multicollinearity. To test the multicollinearity, normal standard is rule of thumb, VIF ≥10 for the definite multicollinearity. In our data, however, all the independent variables had VIF <5 and the tolerance is higher than the threshold level that is >0.25 (see Table 7). Showing data is safe from the multicollinearity threat.

**Table 7:** Multicollinearity.

**Independent variables**	**Tolerance**	**VIF**
VRS	0.38	2.65
MH	0.51	1.95
PA	0.49	2.04

## Results

### Model fit

In the current study, we formed the four constructs in order to measure the model fit indices (68). In our model, we included mental health and performance anxiety as mediators between. Virtual reality sporting experiences and sports endurance performance. The results of the proposed model were good (see Table 8).

**Table 8:** Model fit indices.

	**Model fit**								
Model	Description of model	χ^2^	Df	χ^2^/df	GFI	NFI	TLI	CFI	RMSEA
Model 2	Multi mediation	243.45	99	2.46	0.95	0.86	0.97	0.98	0.05

### Model testing

**Figure 1:**
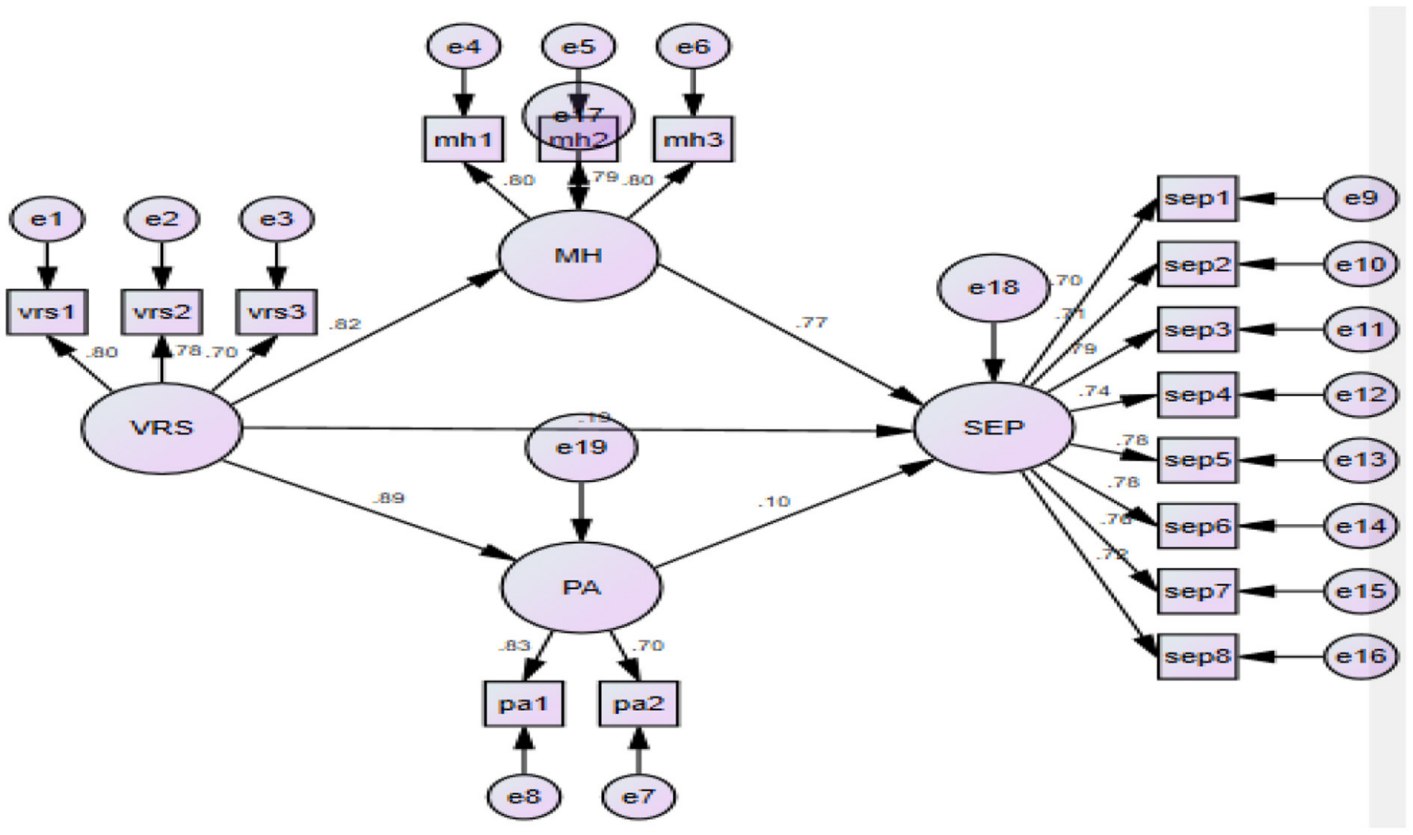
Structural model.

We tested the direct and indirect effects of virtual reality sporting experience on the sports enduring performance with the help of structural equation modeling (Figure 1). Moreover, the indirect effects *via* mental health and the performance anxiety were compared.

A mediation model (see Table 9) produced a good fit with the data (χ^2^ = 243.45, df = 99 χ^2^/df = 2.46 GFI = 0.95, CFI = 0.98, TLI = 0.97, NFI = 0.86, RMSEA = 0.05), suggesting that virtual reality sporting experience significantly effect the dependent variable i.e., sports enduring performance.

**Table 9:** Direct and indirect effects.

**Independent variable/ dependent variable**	**Mental health**	**Performance anxiety**	**Sports endurance performance**		
	**Direct effect**	**Direct effect**	**Direct effect**	**Indirect effect *via* MH**	**Indirect effect *via* PA**	**Comment**
Virtual reality sporting experience	0.81^**^	0.89^**^	0.18	0.63^**^	0.08^*^	Full mediation
Mental health	—	—	0.77^**^	—	—	
Performance anxiety	—	—	0.10^*^	—	—	

* Significant at 5%.

** Significant at 1%.

Virtual reality sporting experiences significantly positively affect mental health (i.e., 0.81 at 5 and 1%). Similarly, virtual reality sporting experiences reduce performance anxiety significantly (i.e., 0.89 at 5 and 1%). Mental health has a significant positive effect on sports endurance performance (i.e., 0.77 at 5 and 1%). Performance anxiety has a positive and significant effect on sports endurance performance (i.e., 0.10 at 5%). effect of virtual reality sporting experiences on sports enduring performance is insignificant, while the indirect effect *via* mental health is positive and significant (i.e., 0.63 at 5 and 1%). The indirect effect of virtual reality sporting experiences on sports enduring performance *via* performance anxiety is significant and positive (i.e., 0.08 at 5%). The findings show that mental health and performance anxiety fully mediate the relationship between virtual reality sporting experiences and long-term performance in sports. Also, the effect that comes from mental health is stronger than the effect that comes from performance anxiety.

## Discussion

Virtual reality sporting experiences significantly positively affect mental health (i.e., 0.81 at 5 and 1%). Similarly, virtual reality sporting experiences reduce performance anxiety significantly (i.e., 0.89 at 5 and 1%). Mental health has a significant positive effect on sports endurance performance (i.e., 0.77 at 5 and 1%). Performance anxiety has a positive and significant effect on sports endurance performance (i.e., 0.10 at 5%). The effect of virtual reality sporting experiences on sports enduring performance is insignificant, while the indirect effect *via* mental health is positive and significant (i.e., 0.63 at 5 and 1%). The indirect effect of virtual reality sporting experiences on sports enduring performance *via* performance anxiety is significant and positive (i.e., 0.08 at 5%). The findings show that mental health and performance anxiety fully mediate the relationship between virtual reality sporting experiences and long-term performance in sports. Also, the effect that comes from mental health is stronger than the effect that comes from performance anxiety.Participation in a virtual reality athletic experience may provide significant therapeutic benefits for one's mental health. Both the theory of social comparison and the hypothesis of social facilitation may be used to explain the results, and both can be used to demonstrate how the results are consistent with their respective assumptions. Both the theory of social facilitation and the theory of social comparison are acceptable avenues of inquiry that might be explored to address the question of why some individuals do better in the presence of others. However, such arguments should virtually never be used in any situation unless the individual has no other option. It has been shown that the mental health of an athlete has a positive and substantial effect on their long-term performance.

The results provide validity to the argument that a competitor's degree of difficulty in a VR-based competitive environment has the ability to affect performance. In other words, the amount of difficulty offered by a rival in a VR-based competitive environment has the ability to affect not just performance but also the outcome of the competition. The outcomes give evidence in support of this position. People have a natural inclination to assess their own performance in relation to the performance of others in their surrounding environment, consistent with the social comparison hypothesis. People prefer to judge their own performance relative to the performance of others, which is consistent with this tendency. The bulk of these performance declines may be linked to mental tiredness, which, according to the area of study known as sports physiology, is the outcome of high-intensity persistent mental activity (44). According to the psychological model of endurance performance (45), which states that perception of effort is the “cardinal exercise stopper” (46), the perception of effort during the performance of a physical task can be affected by a number of factors, including the induction of mental fatigue beforehand. In other words, mental tiredness produced prior to the performance of a physical activity might influence the perception of effort throughout its execution. In other words, the mental fatigue that precedes the performance of a physical action may influence how much effort is seen to be spent during the actual execution of the activity. Anxiety about an upcoming performance may have a substantial and positive effect on an athlete's performance over their whole career. The direct impact of virtual reality sports training on athletes' long-term performance is negligible; but, the indirect effect, which is beneficial to mental health and substantial in and of itself, is an entirely different story. By reducing athletes' levels of performance anxiety, virtual reality sports experiences have a large and favorable indirect effect on their long-term performance. This is a direct result of the sports simulation in virtual reality. According to the findings, the association between having a virtual reality athletic experience and having a long-lasting performance in sports mediates the relationship between mental health and performance anxiety. In addition, the effect of increases in mental health is greater than that of reductions in performance anxiety. This is because the magnitude of the indirect benefit generated by these modifications is greater.

### Theoretical implications

This research contributes to the theoretical understanding of Chinese athletes' endurance performance and the influence of virtual reality sports experience on performance enhancement. This is one of the first studies to look into what causes Chinese athletes to be good at endurance sports and how virtual reality experiences can help improve their sporting performance. Both the theory of social comparison and the social facilitation are used to explain the findings, as well as how the results are compatible with their respective hypotheses. The theory of social facilitation and the theory of social comparison are viable pathways that answer the issue of why some people do better in the company of others. The findings imply that during the athlete's social lives, they all compare themselves to others, whether they are comparing their appearance to that of media celebrities or their talents to the sporting idols. This implication is again in line with the proposition of social comparison theory which is a psychological explanation for our tendency to make comparisons between ourselves and others.

This study attempts to identify, assemble, and evaluate the most compelling evidence in order to improve outcomes related to anxiety and depression. This endeavor aims to provide the framework for future research in this area. The advancement of VR enables the creation of interactive activities. VR workout games may increase motivation, promote physical activity and aid in physical rehabilitation, according to prior research. Virtual reality (VR) exercise has the potential to 1 day be used to treat mental health conditions such as anxiety and depression. This is primarily attributable to the fact that virtual reality (VR) devices may now be coupled to traditional pieces of exercise equipment like treadmills and cycles. Unfortunately, very little study has been done on the influence of virtual reality (VR) exercise on the outcomes associated to anxiety and depression. In light of this, the objective of this study is to conduct an analysis of the data already collected about the impact that virtual reality (VR) exercise has on mental health conditions such as anxiety and depression.

The findings of the study further imply that combining VR with exercise equipment (VR exercise), according to research and expert opinion, has the potential to boost the psychological benefits of exercise and the likelihood of long-term exercise adherence. VirZoom, for instance, is a VR exercise bike compatible with the vast majority of VR headsets (such as the Oculus Rift, PlayStation VR, Samsung Gear VR, HTC Vive, and so on). This enables academics and medical providers to use VR exercise to enhance patients' health. Both sadness and anxiety have been found to benefit from physical activity. Given the pleasurable aspects of currently available VR exercise games and the favorable benefits that exercise has been shown to have on anxiety and depression, VR exercise may be regarded as a potentially effective strategy for alleviating anxiety and depression symptoms.

### Practical implications

Virtual Reality, according to the Ministry of Industry and Information Technology, has the potential to drastically impact the lives of individuals who encounter it. China has a potential to join this booming business and become a global leader in the development of VR technology, since virtual reality is only starting to develop on a global scale and the technology is not yet fully developed (it requires appropriate hardware and content). Notable is the fact that the Chinese government uses “Virtual Reality” as an umbrella name for all immersive technologies (VR, AR, and MR), but we constantly use other terms such as “XR.” The findings led the Chinese government to provide athletes with enough institutional support. The outcomes of the research may aid athletes by enhancing their parenting abilities and performance. Numerous tales of athletes experiencing mental stigma have recently resurfaced in the media; hence, the present research may also be applicable to similar situations. Policymakers can also build a system to help with virtual reality sports experiences.

Some responders said that they were coping with mental health concerns, which impacted their endurance performance. The expertise gained from the virtual reality exercises and applications is assisting them in maintaining the current performance standards and achieving the desired standards. In addition, a number of Chinese respondents have said that performance anxiety is a crucial aspect in understanding the link between virtual reality experience and endurance performance. In accordance with the opinions expressed by respondents and the findings of the current study, it can be inferred that athletes can better plan their virtual reality experience to prevent and minimize the various forms of mental health issues and performance deficits they may experience while engaging in sports activity. Thus, one of the roles of policymakers is to create a system that provides players with the tools they need to maximize their virtual sports experience.

It is now impossible to compete well in modern sports without making use of some kind of technological aid, particularly for athletes. The government of China has already devised a strategy to make the country the undisputed leader in virtual reality by the year 2025. China's goal is to become the industry leader in virtual reality (VR) patents, standards, and products. It has the goal of establishing a flourishing ecosystem that includes Chinese businesses that are competitive on a global scale and a developed infrastructure for the production of virtual reality (VR) products. Because of this, the government will be interested in the results of this research, and researchers will stress the importance of virtual reality athletic experience and how it affects endurance performance.

### Limitations and future directions

When analyzing the study's findings, it is important to take into consideration many limitations. Future studies may apply a longitudinal data collection technique in order to expand the scope of their research, as opposed to the present study, which used a survey questionnaire method using cross-sectional data collected at a certain period. Since all research components were assessed using a single survey instrument, there was a risk of common method variance since the current study relied on self-reported questionnaires from respondents. CMV makes it difficult to analyze psychological data, especially human perspectives, gathered from study participants within a certain time frame. The data gathered for the independent, dependent, and mediator variables in this study were impacted by people's viewpoints. Future research may use strategies based on objective assessment measures to reduce common process variance. Additionally, since the group's membership was so young, we focused on it, which may have restricted generalizability and application.

Since the present study only analyzed the virtual sport experience of the participants, future research should focus on the audience as well as other key variables when determining the purpose of virtual reality in sports. According to Brighton, one must place equal weight on the attention of the audience. Within this virtual sports arena, according to Shi, who is the senior product marketing manager at Agora, fans will have the option to create their own sports avatars, purchase sports equipment, engage in social activities, co-watch events, throw parties, practice, work, and play games. It would lead to a watching experience that was both more vivid and creative as a consequence. People will be able to participate in leagues and interact with various items, and the metaverse will have a big effect on the practice of sports. When it comes to the metaverse, sports will be able to make the most of their intellectual property. In subsequent research, cognitive and social aspects could also be included.

## Conclusion

The main purpose of this study is to examine the effects of VR sports on the endurance performance of Chinese athletes. It was also looked at how the athletes' mental health and performance anxiety mediated the relationship between VR sports experience and Chinese athletes' endurance performance. The young Chinese athletes who are taking part in this study are multi-sport competitors. This group of youthful athletes is a perfect reflection of the state of modern sports. Convenience sampling was used to choose a sample from the total population that is reflective of the results of this research. Among the total of 400 surveys we received, only 145 were usable due to incomplete responses. As an added measure, a statistical evaluation of the sample size was performed using the G^*^Power version 3 software. The data was analyzed using SEM-AMOS, a statistical tool. The survey was designed using a Likert scale, so respondents could select one of five options. Mental health and performance anxiety are the only moderators of the relationship between VR sports experiences and athletes' long-term performance. Furthermore, one's mental wellness has a far larger impact than performance anxiety does. These results provide support to the hypothesis that engaging in a virtual reality sports experience might have significant positive effects on one's mental health. The results may be explained by either the social comparison theory or the social facilitation hypothesis, and both can be used to demonstrate that the results are compatible with their respective theories. Social comparison theory and the social facilitation hypothesis both account for the data. Why some individuals do better in the presence of others may be explained by studying either the theory of social facilitation or the theory of social comparison. However, these kinds of justifications should never be used until absolutely no other options can be found. Mental wellness has been found to have a significant and positive effect on an athlete's long-term performance. These results provide credence to the idea that a competitor's skill level in a virtual reality competition might be affected by the level of difficulty of the competition. To rephrase, in a VR-based competitive setting, the amount of difficulty a rival presents may affect not just performance but also the final score. These results provide weight to this argument. The results might have positive effects on players' parenting and performance. Furthermore, governments may set up a system to allow for VR sports contests.

## Data availability statement

The original contributions presented in the study are included in the article/supplementary material, further inquiries can be directed to the corresponding author.

## Ethics statement

The study was reviewed and approved by the Institutional Research Ethical Committee for the School of Physical Education at Jiaying University, China. Written informed consent was obtained from all participants who had volunteered to participate in this study.

## Author contributions

ZH, D-HC, BL, and ZL were involved in the conception and design of conceptualization, findings interpretation, and review and finalization of the paper. HT and ZL were involved in data analyses, interpretation, and review and finalization of the findings. All authors have given final approval of the version to be published.

## Funding

This work was supported by the National Social Science Fund of China under Grant No. 20BTY079.

## Conflict of interest

The authors declare that the research was conducted in the absence of any commercial or financial relationships that could be construed as a potential conflict of interest.

## Publisher's note

All claims expressed in this article are solely those of the authors and do not necessarily represent those of their affiliated organizations, or those of the publisher, the editors and the reviewers. Any product that may be evaluated in this article, or claim that may be made by its manufacturer, is not guaranteed or endorsed by the publisher.
